# Loss of Actin-Based Motility Impairs Ectromelia Virus Release In Vitro but Is Not Critical to Spread In Vivo

**DOI:** 10.3390/v10030111

**Published:** 2018-03-05

**Authors:** Melanie Laura Duncan, Jacquelyn Horsington, Preethi Eldi, Zahrah Al Rumaih, Gunasegaran Karupiah, Timothy P. Newsome

**Affiliations:** 1 School of Life and Environmental Sciences, The University of Sydney, Sydney, NSW 2006, Australia; melanie.duncan@sydney.edu.au (M.L.D.); jacquelynjh@gmail.com (J.H.); 2 School of Medicine, College of Health and Medicine, The University of Tasmania, Hobart, TAS 7005, Australia; preethi.eldi@gmail.com (P.E.); rumaihxj@gmail.com (Z.A.R.); guna.karupiah@utas.edu.au (G.K.); 3 The John Curtin School of Medical Research, Australian National University, Canberra, ACT 2601, Australia

**Keywords:** actin-based motility, viral release, A36, Arp2/3 complex, virus transport

## Abstract

Ectromelia virus (ECTV) is an orthopoxvirus and the causative agent of mousepox. Like other poxviruses such as variola virus (agent of smallpox), monkeypox virus and vaccinia virus (the live vaccine for smallpox), ECTV promotes actin-nucleation at the surface of infected cells during virus release. Homologs of the viral protein A36 mediate this function through phosphorylation of one or two tyrosine residues that ultimately recruit the cellular Arp2/3 actin-nucleating complex. A36 also functions in the intracellular trafficking of virus mediated by kinesin-1. Here, we describe the generation of a recombinant ECTV that is specifically disrupted in actin-based motility allowing us to examine the role of this transport step in vivo for the first time. We show that actin-based motility has a critical role in promoting the release of virus from infected cells in vitro but plays a minor role in virus spread in vivo. It is likely that loss of microtubule-dependent transport is a major factor for the attenuation observed when *A36R* is deleted.

## 1. Introduction

Ectromelia virus (ECTV) is an orthopoxvirus endemic to mice that has been used extensively as a model for variola virus, the causative agent of smallpox [1]. In common with other orthopoxviruses, ECTV is among a select group of bacterial and viral pathogens able to harness actin-nucleating activity at the pathogen surface to promote their spread [2,3,4]. During infection of host cells, this activity provides a propulsive force that can accelerate microbes through the host cytosol (*Listeria monocytogenes*; *Autographa californica* multiple nucleopolyhedrovirus (AcMNPV)) or across the external surface of the host plasma membrane (vaccinia virus (VACV); enteropathogenic *Echerichia coli* (EPEC)). The contribution of actin-based motility to disease progression is difficult to assess in these complex systems due to the pleiotropy of pathogen-encoded activators of actin polymerization and the lack of availability of in vivo experimental models that faithfully reproduce endogenous infections.

Our understanding of the actin-based motility of orthopoxviruses is primarily derived from VACV, the prototypal poxvirus used as the live vaccine to eradicate smallpox. However, actin-based motility is conserved in the orthopoxvirus genus including variola virus, monkeypox virus, ECTV [5] and further afield in the poxvirus family such myxoma virus and yaba-like disease virus (YLDV) [6,7]. Replication of VACV leads to production of two mature, infectious morphological variants: mature virus (MV), with a single membrane derived from the endoplasmic reticulum; and wrapped virus (WV), which possess one or two additional membranes of a trans-Golgi network (TGN) origin [8,9,10]. Once WV are formed at a perinuclear site, they are translocated to the cell periphery by the action of A36, F12, and E2, which collectively stabilize the microtubule motor complex, kinesin-1, at the viral surface [11,12]. The cytoplasmic domain of A36 contains two Trp-Asp/Trp-Glu (WD/WE) motifs that associate with kinesin-1 via tetratricopeptide repeats on kinesin light chain (KLC) isoform 1 [13,14]. F12 and E2 associate with KLC isoform 2, stabilizing the virus–kinesin-1 association [14]. At the cell membrane, the outer WV membrane fuses with the plasma membrane resulting in cell-associated extracellular virus particles that occupy membrane pits at the cell surface [8]. Tyrosine phosphorylation of the cytoplasmic tail of A36 triggers a signaling cascade culminating in the recruitment and activation of the Arp2/3 complex and robust actin polymerization [15,16]. The activity of the Arp2/3 complex results in WV being released from their membrane pits, which are then readily visualized traversing the cell surface on wakes of F-actin comets.

Actin-based motility of VACV can be ablated by the deletion of *A36R* (the gene that encodes A36) or mutation of the critical tyrosine residues Y112 and Y132 (=VACV-A36^YdF^). As A36 is pleiotropic, only A36^YdF^ specifically disrupts actin-based motility while leaving the kinesin-1 recruitment function intact [15]. Studies with VACV-A36^YdF^ reveal a variety of defects at the cellular level in viral cell-to-cell transmission. Cells infected with VACV-A36^YdF^ do not form actin comets and plaques formed by this virus are significantly reduced in size. Several mechanisms have been proposed to account for how actin-based motility might lead to a defect in cell-to-cell transmission. When actin-based motility localizes to the cell edge, virus-tipped membrane protrusions form that might facilitate infection of adjacent cells [3]. Such a mechanism is thought to facilitate spread of the bacterium *Listeria monocytogenes*, which, following vacuolar escape, spread throughout the cytoplasm on actin comets and also form filopodia extensions laden with bacteria [17,18]. Actin-based motility also promotes repulsion of super-infecting virions whereby early-stage infected cells repel extracellular virus leading to faster spread in a monolayer [19]. Finally, it has been proposed that actin-based motility may be required to disengage WV from the cell surface following exocytosis thereby releasing free virus to the extracellular environment [15].

Infection of mice with VACV lacking A36 clearly reveal an important role in mediating virus spread in an intact host as VACV-ΔA36R exhibits a marked loss in virulence when administered in high doses via the intranasal route [20]. The respective roles of actin-based motility and microtubule-based transport that lead to the attenuation are unknown. The natural host of VACV is also unknown and high titers of virus are required to infect experimental mice. 

We have previously characterized the role of the VACV-*A36R* homolog in ECTV (ECTV-*A36R*) and demonstrated that the key motifs that mediate kinesin-1 recruitment and actin-based motility are conserved [2]. Susceptible BALB/c mice survive infection with high doses of ECTV-ΔA36R but were nonetheless able to raise protective immunity. We have now generated an ECTV recombinant virus, ECTV-A36R^Y112F^ that specifically ablates actin-based motility. In VACV-*A36R*, both Y112 and Y132 contribute to actin-based motility but only the Y112 is necessary and sufficient for robust virus-associated actin nucleation [15,21]. Only the Y112 site is conserved in ECTV-*A36R* and, as expected, cells infected with ECTV-A36R^Y112F^ do not exhibit actin-based motility of WV. Our analysis of ECTV-Moscow (ECTV-Mos, the parental strain and ECTV reference strain, accession AF012825), ECTV-ΔA36R and ECTV-A36R^Y112F^ reveals that actin-based motility does not contribute significantly to plaque size but does have a major role in promoting virus release from infected cells. When virus spread was assayed in C57BL6 mice from subcutaneous injection, ECTV-A36R^Y112F^ was only mildly attenuated in spread to the liver compared with the parental virus; a phenotype that was not exacerbated in interferon γ deficient (IFN-γ^−/−^) or IFN-α/β receptor (R) (IFN-α/βR^−/−^) genetic backgrounds.

## 2. Materials and Methods 

### 2.1. Cells and Viruses

African green monkey kidney cells (BSC-1) and HeLa cells were maintained in Dulbecco’s Modified Eagle’s Medium (DMEM; Invitrogen, Carlsbad, CA, USA) supplemented with 5% fetal bovine serum (FBS), 292 mg/mL l-glutamine, 100 units/mL penicillin and 100 mg/mL streptomycin (DMEM-FPSG) at 37 °C and 5% CO_2_. Viruses used in this study are ECTV-Mos (GenBank accession no. AF012825), ECTV-ΔA36 which has been described previously [2], from this virus the ECTV-A36^Y112F^ and ECTV-A36^Res^ viruses were created. Briefly, plasmids containing recombination cassettes to replace the mCherry fluorescent protein and guanosine phosphotransferase (GPT) selection markers were made with the *A36R* gene either with the point mutation (for ECTV-A36^Y112F^) or parental sequence (for ECTV-A36^Res^) and approximately 900 bp flanking regions of homologous ECTV genomic DNA adjacent to the *A36R* locus. Constructs were transfected according to manufacturer’s instructions (Lipofectamine 2000 reagent, Invitrogen) into HeLa cells infected with ECTV-ΔA36 and homologous recombination allowed to proceed for 24 h. Cells were scraped and the resulting virus screened by loss of GPT resistance and fluorescence as described previously [22]. Viruses were purified by three rounds of plaque purification, and insertion of the correct sequence was confirmed by sequencing.

### 2.2. Plaque Assays

BSC-1 cells were seeded in six-well plates and grown to confluence. Viruses were diluted in serum free DMEM (SFM) and approximately 25 plaque forming units (PFU) was added to each well. After incubation at 37 °C in 5% CO_2_ for 1 h, the cells were washed and overlaid with 1.5% carboxymethyl cellulose (CMC) in minimal essential medium (MEM) containing 2.5% FBS, 292 mg/mL l-glutamine, 100 units/mL penicillin and 100 mg/mL streptomycin. Cells were incubated for 6 days post infection (days pi) and then the overlay removed and cells stained with 1% crystal violet in methanol for visualization. Plaque diameter was measured by taking a straight line across the widest point of the plaque using Image J software (version 2.0.0-rc-49/1.51a, National Institutes of Health, Bethesda, MD, USA).

### 2.3. WV Release Assay

Six-well dishes were seeded with BSC-1 cells and incubated with virus (in triplicate) at an multiplicity of infection (MOI) of 0.1 for 1 h. Cells were then washed twice with PBS and DMEM-FPSG was added. The supernatants were collected at 24 h post infection (hpi), and spun at 10,000 rpm for 10 min at 4 °C to remove cells and cellular debris. To quantify the infectious WV, plaque assays of 10-fold serial dilutions of the supernatant were performed on BSC-1 cells as described above. After 6 days, cells were stained with methanol/1% crystal violet and plaques enumerated. All WV assays were performed on at least three separate occasions. 

### 2.4. Immunofluorescence Assay

Cells were grown on glass coverslips, infected with virus, and fixed at 16 or 24 hpi with 3% paraformaldehyde (PFA) in cytoskeletal buffer (CB) [10 mM 2-(N-morpholino) ethanesulfonic acid (MES) buffer, 0.15 M NaCl, 5 mM ethylene glycol-bis(β-aminoethyl ether)-N,N,N′,N′-tetraacetic acid (EGTA), 5 mM MgCl2, 50 mM glucose, pH 6.1] for 10 min at room temperature or ice-cold 100% methanol for 15 min. Cells were blocked in blocking buffer (1% bovine serum albumin (BSA) and 2% FBS in CB) for 20 min and then incubated for 40 min with suitable primary antibodies diluted in blocking buffer. Primary antibodies used in this study were: anti-A36-Y112 [2] and anti-B5 [23]. Fluorescent chemicals used were: Alexa Fluor 568-phalloidin (Invitrogen) and DAPI (4′,6-diamidino-2-phenylindole; Sigma-Aldrich, Saint Louis, MO, USA). After three washes with PBS, secondary antibodies (Alexa Fluor 488-conjugated goat anti-rat IgG and Alexa Fluor 568-conjugated goat anti-rabbit IgG; Invitrogen) diluted in blocking buffer were applied to cells for 20 min. The coverslips were mounted on a glass slide with 0.3–1% (*w*/*v*) p-phenylenediamine (Sigma-Aldrich) in Mowiol mounting medium (10% (*w*/*v*) polyvinyl alcohol 4–88 (Sigma-Aldrich), 25% (*w*/*v*) glycerol, 0.1 M Tris, pH 8.5). Images were captured on an Olympus BX51 microscope (Tokyo, Japan) with a reflected fluorescence system using AnalySIS LS Starter (Olympus Soft Imaging Systems, ver. 2.8).

### 2.5. Immunoblot Analysis

BSC-1 cells were infected with virus at an MOI of 1 for 24 h. Infected cells were harvested in sodium dodecyl sulfate (SDS)-reducing sample buffer (62.5 mM Tris-HCl, 0.25 M glycerol, 2% SDS, 0.01% (*w*/*v*) bromophenol blue, 12.5% (*v/v*) β-mercaptoethanol) and boiled at 95 °C for 5 min. Proteins were separated by SDS-polyacrylamide gel electrophoresis (SDS-PAGE) (resolving gel of 10% acrylamide-Bis solution (37.5:1), 0.375 M Tris-HCl, pH 8.8, 0.1% (*w*/*v*) SDS, 0.1% ammonium persulfate (APS), and 0.1% *N*,*N*,*N*=,*N*=-tetramethylethylenediamine (TEMED); stacking gel of 4% to 30% acrylamide-Bis solution (37.5:1), 0.375 M Tris-HCl, pH 6.8, 0.1% (*w*/*v*) SDS, 0.1% APS, and 0.1% TEMED). Resolved proteins from SDS-PAGE were transferred to nitrocellulose membranes (Hybond-C Extra; Amersham Biosciences, Little Chalfont, UK) and probed with primary antibodies diluted in PBST-milk (5% (*w*/*v*) skim milk in PBS with 0.1% Tween 20). Primary antibodies used in this study were anti-A36-Y112 [2], anti-L1 (NR-631, BEI Resources, Manassas, VA, USA), and anti-β-actin (AC-74, Sigma-Aldrich). The membrane was washed three times in PBST-milk and probed with secondary antibodies conjugated with horseradish peroxidase (HRP) (goat anti-mouse IgG HRP and goat anti-rabbit IgG HRP; Santa Cruz Biotechnology, Dallas, TX, USA). Immunoreactive protein bands were visualized with ECL Western blotting reagent (GE Health, Little Chalfont, UK). 

### 2.6. Mouse Experiments

C57BL/6 wild-type, C57BL/6 IFN-γ^−/−^ and C57BL/6 IFN-α/β receptor (R)^−/−^ (IFN-α/βR^−/−^) mice were challenged by injection subcutaneously in the right hind leg (hock) with 10^3^ PFU virus and sacrificed at 5 days pi. Viral loads in livers, spleens, and lymph nodes were quantified by viral plaque assay on BSC-1 cell monolayers as described above and are expressed as log_10_ PFU per gram of tissue or per popliteal lymph node. 

### 2.7. Ethics Statement

This study was performed in accordance with the recommendations in the Australian code of practice for the care and use of animals for scientific purposes and the Australian National Health and Medical Research Council Guidelines and Policies on Animal Ethics. The Australian National University Animal Ethics and Experimentation Committee approved all animal experiments (Protocol Numbers: J.IG.75.09 and A2012/041). Tribromoethanol (Avertin, Millipore Sigma, Saint Louis, MO, USA) was used as the anesthetic (200–240 mg/kg body weight) given via intra-peritoneal injection prior to infection with virus. The respiration rate of the animals was monitored during anesthesia and recovery took place upon a warm table. Animals were euthanized by cervical dislocation. The animal ethics approval did not allow for mortality experiments and for all mice to be euthanized in the event that 20% of the mice succumbed to disease. For these reasons, all mice were euthanized at Day 5 post-infection.

## 3. Results

### 3.1. Generation of ECTV-A36^Y112F^

**Figure 1:**
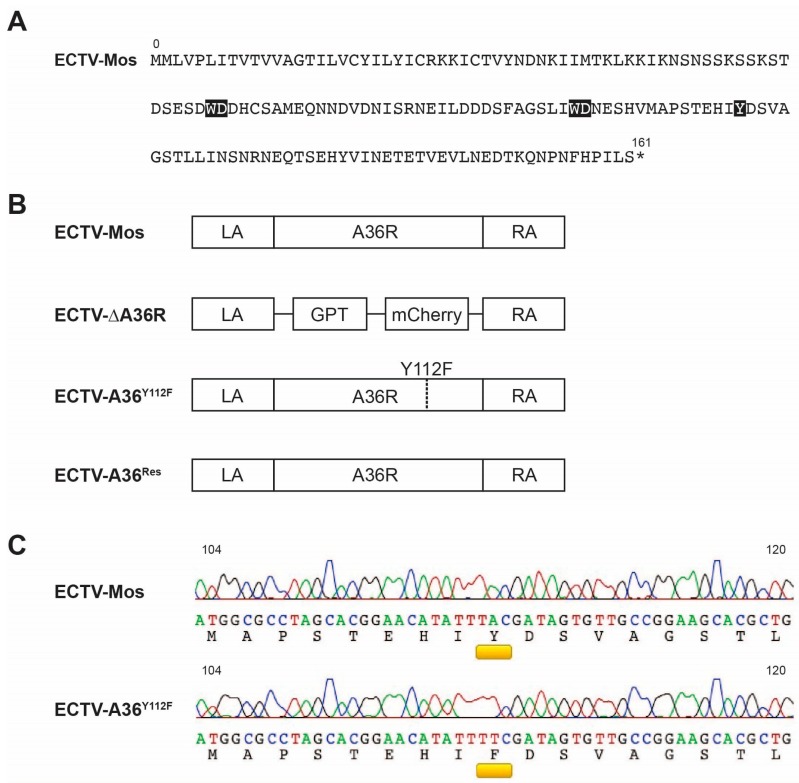
Schematic of Ectromelia virus (ECTV) A36 mutants: (**A**) Translation of the *A36R* open frame in ECTV-Moscow (ECTV-Mos). Highlighted are the Trp-Asp (WD) motifs for binding of kinesin-1 and the putative tyrosine phosphorylation site at position 112. (**B**) Schematic of the four ECTV viruses used in this study: the parental ECTV-Mos, ECTV-ΔA36, ECTV-A36^Y112F^, and ECTV-A36^Res^. (**C**) Sequencing reads of the *A36R* gene of the parental ECTV-Mos and mutated ECTV-A36^Y112F^. Amino acid position 112 highlighted in yellow.

To assess the role of actin-based transport in ECTV, four viruses were used. The parental ECTV-Mos shares a high level of sequence identity with VACV Western Reserve (VACV-WR), with the exception of a large truncation at the C-terminal that removes three Asn-Pro-Phe (NPF) motifs that recruit intersectin-1 [24]. Although the WD/WE motifs that mediate kinesin-1 binding are intact, of the two critical tyrosines in VACV-WR at positions 112 and 132, only the first, Y112, is present in ECTV-Mos (Figure 1A). The *A36R* gene was deleted in ECTV-Mos by replacing the gene with selectable (GPT resistance) and screenable (mCherry fluorescence) markers. This created the ΔA36 virus, which is deficient in both microtubule transport and actin-based motility [2]. From an ECTV-ΔA36 background, ECTV-A36^Res^ and ECTV-A36R^Y112F^ were made by rescue of the small plaque phenotype. ECTV-A36^Res^ restored a wild type copy of *A36R* and was expected to fully restore function of virus. ECTV-A36^Y112F^ carries a Y112F substitution, which was predicted to specifically disrupt the actin-based motility function of *A36R* while leaving other functions intact (Figure 1B). Viruses were then sequenced at the *A36R* gene by Sanger sequencing to confirm the mutation (Figure 1C). 

**Figure 2:**
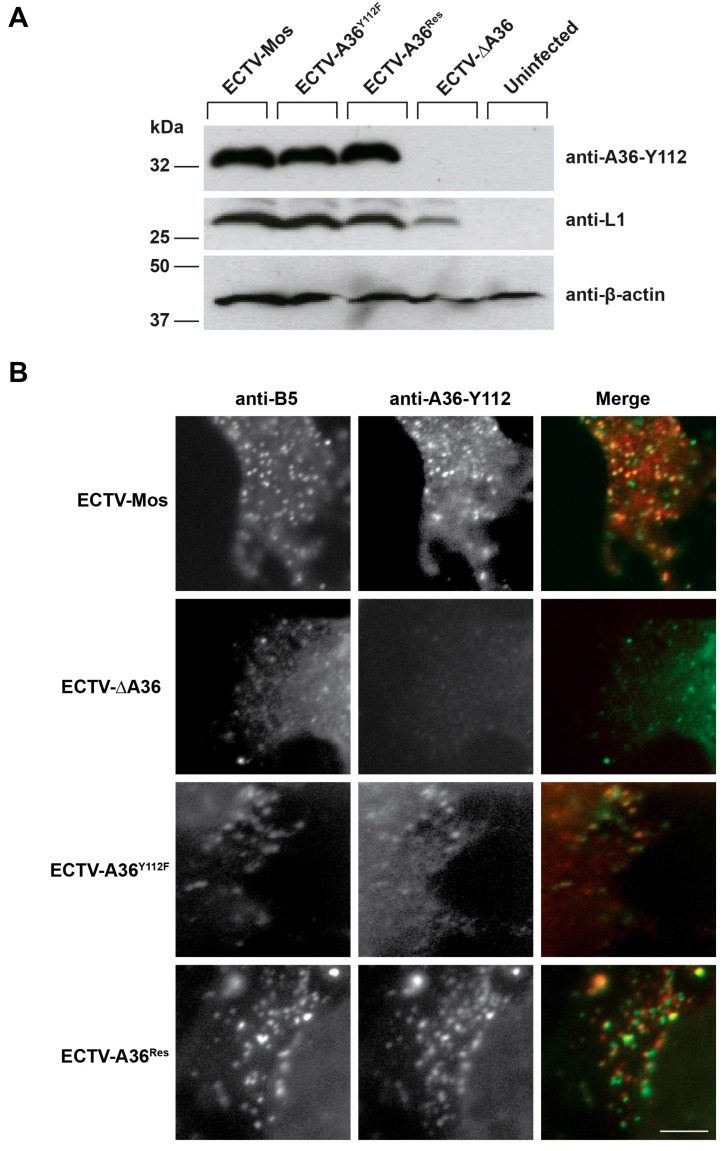
ECTV-A36^Res^ has normal A36 expression and localization. (**A**) BSC-1 cells were infected at a multiplicity of infection (MOI) of 1, and cell lysates were collected at 48 hours post infections (hpi). Cell lysates were separated by sodium dodecyl sulfate-polyacrylamide gel electrophoresis (SDS-PAGE) and transferred to nitrocellulose membranes for immunoblotting with anti-A36-Y112, anti-L1 and anti-β-actin. Molecular size markers are indicated on the left. (**B**) HeLa cells were infected with the indicated viruses and fixed at 24 hpi and probed with anti-B5 (green) and anti-A36-Y112 (red) and visualized by immunofluorescence microscopy. Scale bar: 5 μm.

To analyze A36 expression (the protein encoded by the *A36R* gene), cells were infected with ECTV-Mos, ECTV-ΔA36, ECTV-A36^Y112F^ and ECTV-A36^Res^ and the lysates collected at 48 h post infection (hpi). A36 was detected in ECTV-Mos, ECTV-A36^Y112F^ and ECTV-A36^Res^ at the expected size of 34 kDa (Figure 2A). We therefore successfully confirmed ECTV-A36^Res^ restores normal levels of A36 expression and that the Y112F mutation does not grossly disrupt A36 stability or mobility. We further analyzed A36 localization by immunofluorescence assay. Cells were infected with ECTV-Mos, ECTV-ΔA36, ECTV-A36^Y112F^ and ECTV-A36^Res^ and fixed at 16 hpi. Cells were stained with antibodies against A36 and known WV protein B5. As expected, A36 colocalized with B5 in ECTV-Mos, ECTV-A36^Y112F^ and ECTV-A36^Res^, but not in ECTV-ΔA36, where it is only detected at background levels (Figure 2B). This confirms that both ECTV-A36^Y112F^ and ECTV-A36^Res^ viruses restore normal A36 expression and localization.

### 3.2. A36R Is Required for ECTV Actin-Based Motility and Virus Release

**Figure 3:**
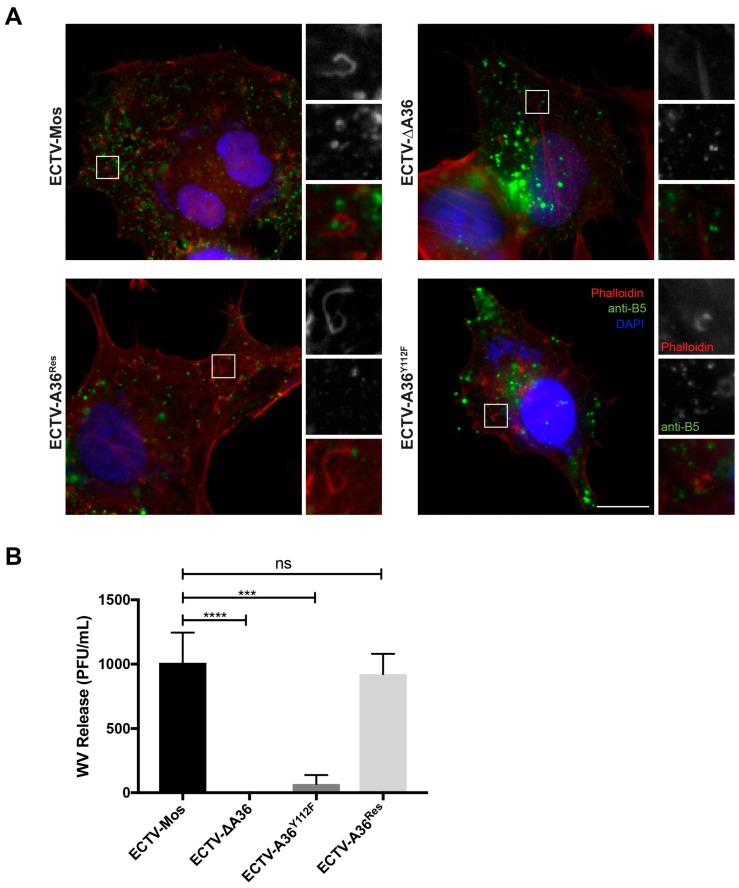
Release of WV is reduced in the absence of actin-based motility. (**A**) HeLa cells were infected with the indicated viruses and fixed at 16 hpi, and probed with anti-B5 (green), phalliodin (red), and DAPI (4′,6-diamidino-2-phenylindole) (blue) and visualized by immunofluorescence microscopy. Scale bar: 10 μm. (**B**) BSC-1 cells were infected at a multiplicity of infection (MOI) of 0.1 and supernatants collected at 24 hpi and centrifuged at 10,000 rpm for 10 min to remove cells and cellular debris. Plaque assays were then performed in BSC-1 cells infected with serially diluted supernatant and overlaid with 1.5% carboxymethyl cellulose in minimal essential medium (CMC-MEM) and stained at six days pi with crystal violet to quantify plaques to determine viral titre. Data for three experimental replicates were pooled. (**** *p* < 0.0001; *** *p* < 0.001; ns, non-significant; unpaired *t*-test).

We then tested the ability of ECTV-A36^Y112F^ to undergo actin-based motility. HeLa cells were infected with ECTV-Mos, ECTV-ΔA36, ECTV-A36^Y112F^ and ECTV-A36^Res^ and fixed at 16 hpi, then stained with anti-B5R to visualize wrapped virus and phalloidin to visualize F-actin. Actin-based motility is evident in ECTV-infected cells by the association of WV with comets of F-actin, which is absent when *A36R* has been deleted (Figure 3A). Although small accumulations of F-actin could occasionally be found in association with WV in ECTV-A36^Y112F^-infected cells, no comets were observed, which is consistent with an essential role for Y112F in promoting actin-based motility of ECTV. We have previously shown that release of WV by VACV is strongly attenuated by the Y112F mutation (and also by the Y112F/Y132F double mutation, VACV-A36^YdF^) [15]. In line with these results, virus release by ECTV is also dependent on actin-based motility as ECTV-A36^Y112F^-infected cells displayed a dramatic (15-fold) reduction in WV release (Figure 3B). Wild type ECTV *A36R* fully restored actin-based motility and WV release (Figure 3A,B).

### 3.3. Actin-Based Motility Does Not Contribute to Cell-to-Cell Spread of ECTV

**Figure 4:**
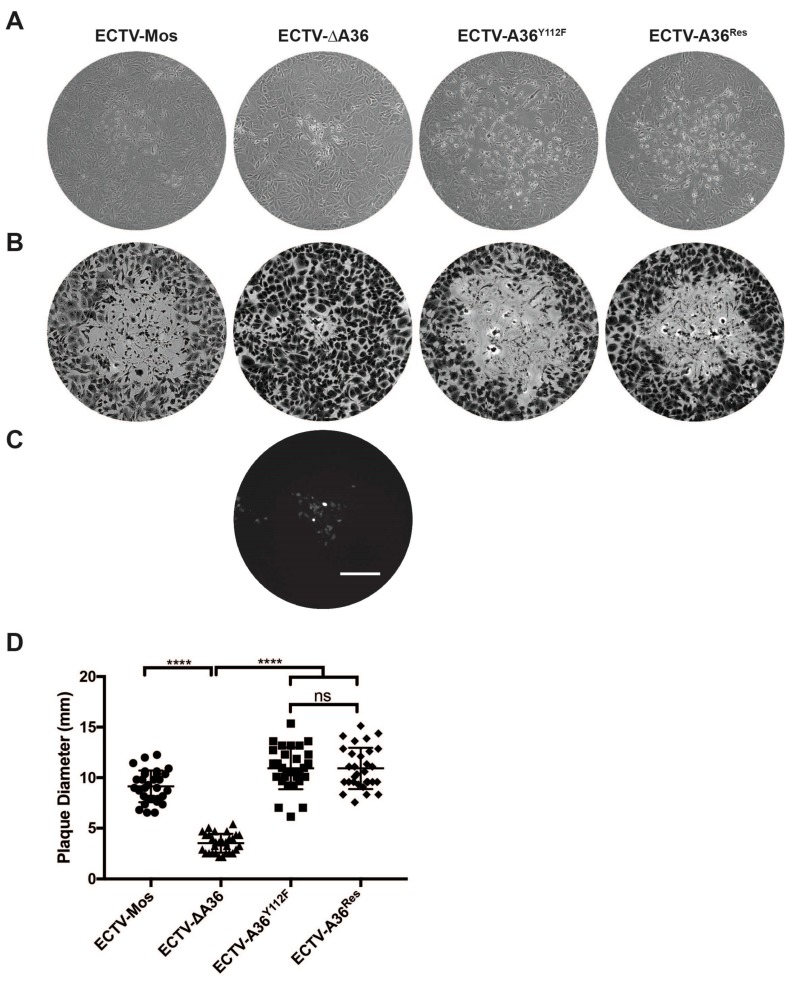
Actin-based motility does not contribute to cell-to-cell spread. Plaque assays were performed in BSC-1 monolayers infected with serially diluted virus and overlaid with 1.5% carboxymethyl cellulose in minimal essential medium (CMC-MEM). At six days pi, plaques were visualized by: phase contrast microscopy (**A**); and fluorescence microscopy for ECTV-ΔA36 (**C**); and then stained with crystal violet (**B**). Scale bar: 200 μm. (**D**) The diameter of each plaque was measured as the widest point across which cytopathic effects were observed. Data for two experimental replicates were pooled (*n* = 30). (**** *p* < 0.0001; ns, non-significant; unpaired *t*-test).

To determine the effect of actin-based motility on cell-to-cell spread of ECTV, plaque assays were performed in BSC-1 cells. Cells were infected with ECTV-Mos, ECTV-ΔA36, ECTV-A36^Y112F^ and ECTV-A36^Res^ and plaques allowed to develop over six days. There were no significant differences in plaque size between ECTV-Mos, ECTV-A36^Y112F^ and ECTV-A36^Res^ although we were able to replicate the small plaque phenotype of ECTV-ΔA36 (Figure 4A,B). This is somewhat surprising as VACV-A36^YdF^ exhibits a significant reduction in cell-to-cell spread [12]. Thus, cell-to-cell spread of ECTV is primarily mediated by microtubule-dependent transport with actin-based motility playing a non-significant role.

### 3.4. Actin-Based Motility Plays a Minor Role in ECTV Spread In Vivo

**Figure 5:**
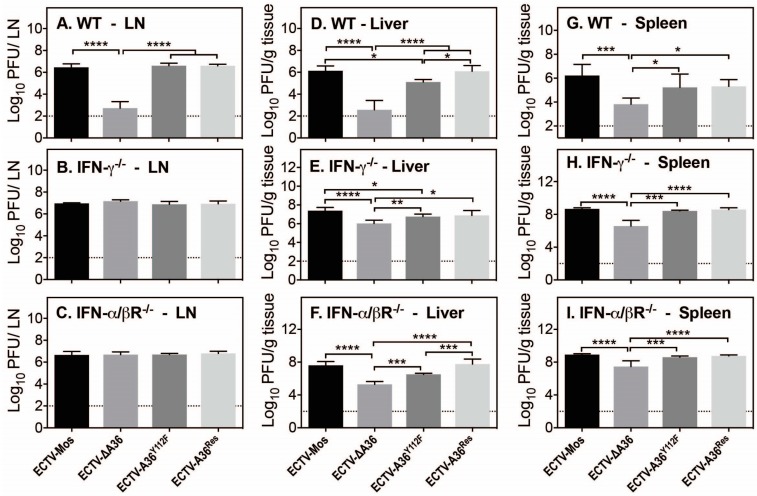
Loss of microtubule transport and actin-based motility results in attenuation in vivo. C57BL/6 mice with WT, IFN-γ^−/−^ or IFN-α/βR^−/−^ genetic backgrounds were infected subcutaneously in the right hind paw with 10^3^ PFU virus and sacrificed at five days pi, viral dissemination to (**A**–**C**) the popliteal lymph nodes; (**D**–**F**) the liver and (**G**–**I**) the spleen, was quantified by plaque assay. (**** *p* < 0.0001; *** *p* < 0.001; ** *p* < 0.01; * *p* < 0.05; one-way ANOVA).

To assess the consequences of loss of microtubule transport and actin-based motility we next examined the ability of ECTV to spread in vivo. ECTV-resistant C57BL/6 mice were infected subcutaneously in the right hind leg with 10^3^ PFU virus and sacrificed five days pi, and viral dissemination to three distal sites (popliteal lymph nodes, liver and spleen) was quantified. To gain further insight into the effects of defective viral transport in vivo, three subsets of mice were used, either WT, IFN-γ^−/−^ or IFN-α/βR^−/−^. Viral dissemination to the lymph nodes was reduced significantly by three orders of magnitude in ΔA36 in WT mice (Figure 5A). However, deletion or mutation of A36 had no discernible effects on viral spread in IFN-γ^−/−^ or IFN-α/βR^−/−^ mice (Figure 5B,C), with ECTV readily disseminating to the lymph nodes in these mice. In contrast, viral spread to the liver was dramatically reduced in ECTV-ΔA36 and significantly reduced in ECTV A36^Y112F^-infected mice (Figure 5D–F). In all murine backgrounds, the change in viral dissemination is greatest for ECTV-ΔA36 infections, while the spread of ECTV-A36^Y112F^ was reduced to a lesser extent. In the spleen, dissemination of ECTV-ΔA36 was significantly reduced but a phenotype for ECTV-A36^Y112F^ was only observed in WT mice (Figure 5G–I). These results suggest that defects in actin-based motility have a minor, but detectable, role in mediating spread of ECTV in vivo but that the primary basis for the attenuation of ECTV-ΔA36 is loss of microtubule-based transport.

## 4. Discussion

Actin-based motility is a highly conserved feature of the orthopoxvirus genus. Virally induced actin nucleation has been demonstrated for VACV, ECTV, variola and monkeypox viruses [2,5,16,25,26,27], and is predicted to be a feature of replication in other members of this genus based on the conservation of the *A36R* gene, with a sequence identity between 81% and 100% for all published sequences [28]. The A36 protein family includes two tyrosines embedded in a Src-family kinase substrate motif, with the exception of ECTV-A36 that contains only the N-terminal tyrosine [29]. Our results confirm that actin-based motility in ECTV is mediated by the A36 protein, and further demonstrate that the 112 tyrosine is the critical residue required for actin nucleation. This is as expected, but represents only the second instance where the basis for actin-based motility has been mapped genetically in a poxvirus [15,16,25,26]. Within the wider Poxviridae family, actin-based motility has been observed in myxomavirus and YLDV [6,7]. YLDV, a member of the yatapoxvirus genus, has been observed to form virus-associated actin tails during infection, yet lacks an obvious *A36R* homolog [7,30]. It was found that YLDV encodes a functional ortholog of *A36R*, termed *YL126*, which appears to promote actin-based motility. Although containing less than 15% amino acid identity to A36, it is found in an analogous genomic loci (*YL124R* and *YL127R* are orthologs of VACV *A35R* and *A37R*, respectively [28,30]), contains five tyrosines that are able to be phosphorylated, and can furthermore restore actin-based motility in ΔA36 VACV infected cells [28]. *YL126* homologs have been identified in the other members of the yatapoxvirus family as well as myxomavirus, and in members of the carpripoxvirus, leporipoxvirus and suipoxvirus families [28]. The conservation of actin-based motility across diverse poxviruses suggests an important role in viral spread across a range of hosts. 

Our results demonstrate that, while release of ECTV WV into the extracellular media is strongly defective in the absence of actin-based motility, loss of this motility has only minor consequences in vivo for the pathogenesis of ECTV. In contrast, the loss of the microtubule-based transport function of A36 results in a significant reduction of viral spread both in vitro and in vivo. We propose that the critical function of A36, in terms of virulence in vivo, lies in translocating WV to the cell surface. Ablation of actin-based motility did not significantly affect the cell-to-cell spread of ECTV in vitro. This result was somewhat unexpected given that in VACV a mutation in actin-based motility via deletion of one or both tyrosines (VACV-A36^Y112F^ or VACV-A36^YdF^) does result in a reduction of cell-to-cell spread [15]. This suggests that though highly conserved, there is some variation among poxviruses in their requirement for actin-based motility during their replication cycle. A plaque assay quantitates the cell-to-cell spread of virus but not the contribution of spread of WV through the extracellular media, which is inhibited by the overlay. For example, treating VACV with the Abl kinase inhibitor imatinib causes substantial reduction in release of WV from infected cells, yet does not lead to a significant reduction in plaque size [31]. A possible explanation for why ablation of actin-based motility in VACV and ECTV results in different outcomes for cell-to-cell spread may be due to the repulsion of super-infecting virions. This process allows viral spread to occur faster than the rate of replication dynamics by means of extracellular WV being blocked from entry to early infected cells via cell surface signaling, promoting their spread to uninfected cells via actin-based motility [19,32]. Repulsion requires the early expression of A36 and A33 at the surface of infected cells and the expression of B5 on the surface of the repelled WV [19,32]. The absence of an observable defect in ECTV-A36^Y112F^ plaques could be attributable to super-repulsion not playing a role in ECTV spread. In this case, the function of actin-based motility may lie solely in promoting release. We have previously suggested that actin-based motility in poxviruses may have evolved to promote WV release; a mechanism later coopted for super-repulsion. Although our data would appear to support this hypothesis, ECTV-*A36R* is still an early expressed gene. Alternatively, the small plaque size of ECTV may mask the contribution of actin-based motility rendering the assay less sensitive to small differences. 

Our results demonstrate only a minor role for actin-based motility for virus spread in vivo. While we observed a detectable reduction in viral titers in distal organs associated with ECTV-A36^Y112F^, this was negligible in comparison to the effect of deleting A36. Our data also establish that intact IFN-γ and IFN-α/β signaling are critical for A36 function, particularly for virus dissemination to the lymph node, but less so for dissemination to the liver and spleen. While our ethics approval did not allow for mortality experiments, based on the viral load in liver and spleen of the IFN-γ^−/−^ and IFN-α/βR^−/−^ mice at Day 5, and based on our experience with the mousepox model, we predict that gene knock-out mice challenged with ECTV-ΔA36 would have survived, whereas those challenged with ECTV-Mos, ECTV-A36^Y112F^ or ECTV-A36^Res^ would have all succumbed to mousepox. We speculate that if orders of magnitude lower doses of virus were used, we may have seen more significant differences between ECTV-Mos and ECTV-A36^Y112F^ or between ECTV-ΔA36 and ECTV-A36^Y112F^. We conclude that viral release mediated by actin-based motility is not a major determinant to the virulence of ECTV. There is little consensus in the literature as to how WV release correlates to poxvirus virulence in vivo. While poxvirus WV release is complex and not fully characterized, several mutations that increase release have been identified. However, these are typically, but not exclusively, associated with a reduction in cell-to-cell spread as measured by plaque assay and these viruses are attenuated in vivo (for example, VACV-B5P^189S^; [33,34,35,36,37]. The IHD-J strain of VACV carries a mutation that results in viral release 40 times higher than WR but forms normal sized plaques [33]. This strain displays lower mortality than WR in a murine infection model; however, when compared to other strains of VACV, there was a positive correlation of WV release with virulence. This suggests that WR, which produces low numbers of WV, may be the exception rather than the rule [38]. Conversely, a comparative study of variola virus strains found a negative correlation between WV release and virulence [39]. While its role in virulence remains cryptic, there are suggestions that high WV release may have a role in increasing the transmission of virus between individuals [38,39,40]. Future studies may examine this potential role for WV release, a context that is largely mechanistically uncharacterized.

Actin-based motility is utilized not only by poxviruses but also by a wide range of bacterial pathogens including species of *Listeria*, *Rickettsia*, *Shigella*, *Mycobacteria*, *Burkholderia*, and EPEC [41,42]. Understanding the molecular basis for actin-based motility can provide clues about both disease pathogenesis and cellular biology. Indeed the study of *Listeria monocytogenes* motility was key to identifying the Arp2/3 complex as an inducer of actin nucleation [18]. In many cases, actin-based motility of pathogens is difficult to study in whole animal models due to multiple or unclear paths of activation, activators with multiple functions, or lack of an endogenous disease model. Many of these bacteria are human pathogens, and study of their interaction with host cellular systems is hindered by species-specific interactions in small animal models. For example, multiple species-specific barriers exist when mice are infected with *L. monocytogenes*, such as the internalin–E-cadherin engagement during entry [17]. ActA, the nucleator of *L. monocytogenes*, is also highly pleiotropic with a role in the aggregation of bacteria [43]. In ECTV, we have an opportunity to study a pathogen in its natural host, whereby we can manipulate not only the gene responsible for actin-based motility but also ablate actin-based motility with the substitution of a single amino acid. Our understanding of how actin-based motility of ECTV affects the spread of virus in vivo may provide clues about disease progression among poxviruses and other pathogens, and help build a more complete picture of cytoskeletal remodelling by microbes.
